# Low-density lipoprotein encapsulated thiosemicarbazone metal complexes is active targeting vehicle for breast, lung, and prostate cancers

**DOI:** 10.1080/10717544.2022.2096713

**Published:** 2022-07-10

**Authors:** Laila Jaragh-Alhadad, Mayada Samir, Terri J. Harford, Sadashiva Karnik

**Affiliations:** a Department of Chemistry, Faculty of Science, Kuwait University, Kuwait, Safat, Kuwait; b Cardiovascular and Metabolic Sciences Department, Cleveland Clinic Lerner Research Institute, Cleveland, Ohio, USA; c Cleveland Clinic Learner College of Medicine, Case Western Reserve University, Cleveland, Ohio, USA

**Keywords:** Thiosemicarbazone metal-ligand complexes, LDL particles, LDL-receptor, drug delivery, MCF, A549, C42

## Abstract

Cancer is a leading cause of death worldwide and affects society in terms of the number of lives lost. Current cancer treatments are based on conventional chemotherapy which is nonspecific in targeting cancer. Therefore, intensive efforts are underway to better target cancer-specific cells while minimizing the side effects on healthy tissues by using LDL particles as active drug delivery vehicles. The goal is to encapsulate anticancer agents thiosemicarbazone metal-ligand complexes into LDL particles to increase the cytotoxic effect of the agent by internalization through LDL receptors into MCF7, A549, and C42 cancer cell lines as segregate models for biological evaluations targeting tubulin. Zeta potential data of LDL-particles encapsulated anticancer agents showed an acceptable diameter range between 66–91 nm and uniform particle morphology. The results showed cell proliferation reduction in all tested cell lines. The IC_50_ values of LDL encapsulated thiosemicarbazone metal-ligand complexes treated with MCF7, A549, and C42 ranged between 1.18–6.61 µM, 1.17–9.66 µM, and 1.01–6.62 µM, respectively. Western blot analysis showed a potent decrease in tubulin expression when the cell lines were treated with LDL particles encapsulated with thiosemicarbazone metal-ligand complexes as anticancer agents. In conclusion, the data provide strong evidence that LDL particles are used as an active drug delivery strategy for cancer therapy.

## Introduction

**Figure 1.**
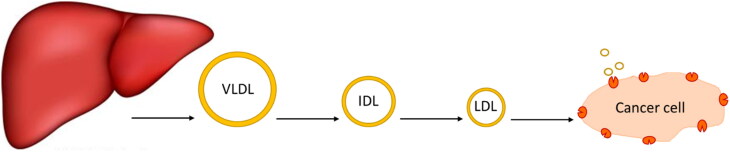
Natural lipids synthesis and their metabolic pathway.

Lipoproteins are a class of complex macromolecules composed of lipid and protein which function to transport lipids in an aqueous environment throughout a systematic circulation (Chung & Wasan, 2004). However, their biological significance in understanding the mechanism of mimicking the native LDL and taking the drug intracellularly provides a novel method in drug delivery strategy (Firestone, 1994; Kader & Pater, 2002; Chung & Wasan, 2004; Lee et al., 2015; Zhang & Huang, 2017). Lipoproteins are classified based on their densities into chylomicron, very-low-density lipoprotein (VLDL), intermediate-density lipoprotein (IDL), and low-density lipoprotein (LDL), and high-density lipoprotein (HDL) (Kader & Pater, 2002; Chung & Wasan, 2004). The liver synthesizes VLDL which sets converted to IDL by lipase enzyme to LDL that is rich in cholesterol and gets distributed to body tissues (Chung & Wasan, 2004) as shown in Figure 1. Various lipoprotein receptors are expressed on the cell surface and regulate lipoprotein uptake by endocytosis (Kader & Pater, 2002; Chung & Wasan, 2004; Nikanjam et al., 2007). The circulating LDL binds to the LDL-receptor through the recognition of apolipoprotein B-100 (Nikanjam et al., 2007; Zhang & Huang, 2017), internalizing through the clathrin-coated vessel (Firestone, 1994), and degradative by lysosomes resulting in transporting cholesterol cargo and use or storing it in the cell (Chung & Wasan, 2004; Lee et al., 2015). Cancer cells have high LDL demand because new dividing cells need cholesterol which is the building block for new cell membranes synthesis (Firestone, 1994; Nikanjam et al., 2007) and has been observed in the colon (Wang et al., 2017), prostate (Stopsack et al., 2017), adrenal (Nikanjam et al., 2007), breast (Gallagher et al., 2017), lung (Li et al., 2020), leukemia (Zhao et al., 2019), brain (Ahmad et al., 2019), and ovarian (Tania et al., 2010) cancers (Chaudhary et al., 2019). Furthermore, several site-specific cancer studies showed the relationship between cholesterol levels and different stages of cancer progression (Chaudhary et al., 2019), which are upregulated with the increase of cancer stages.

**Figure 2.**
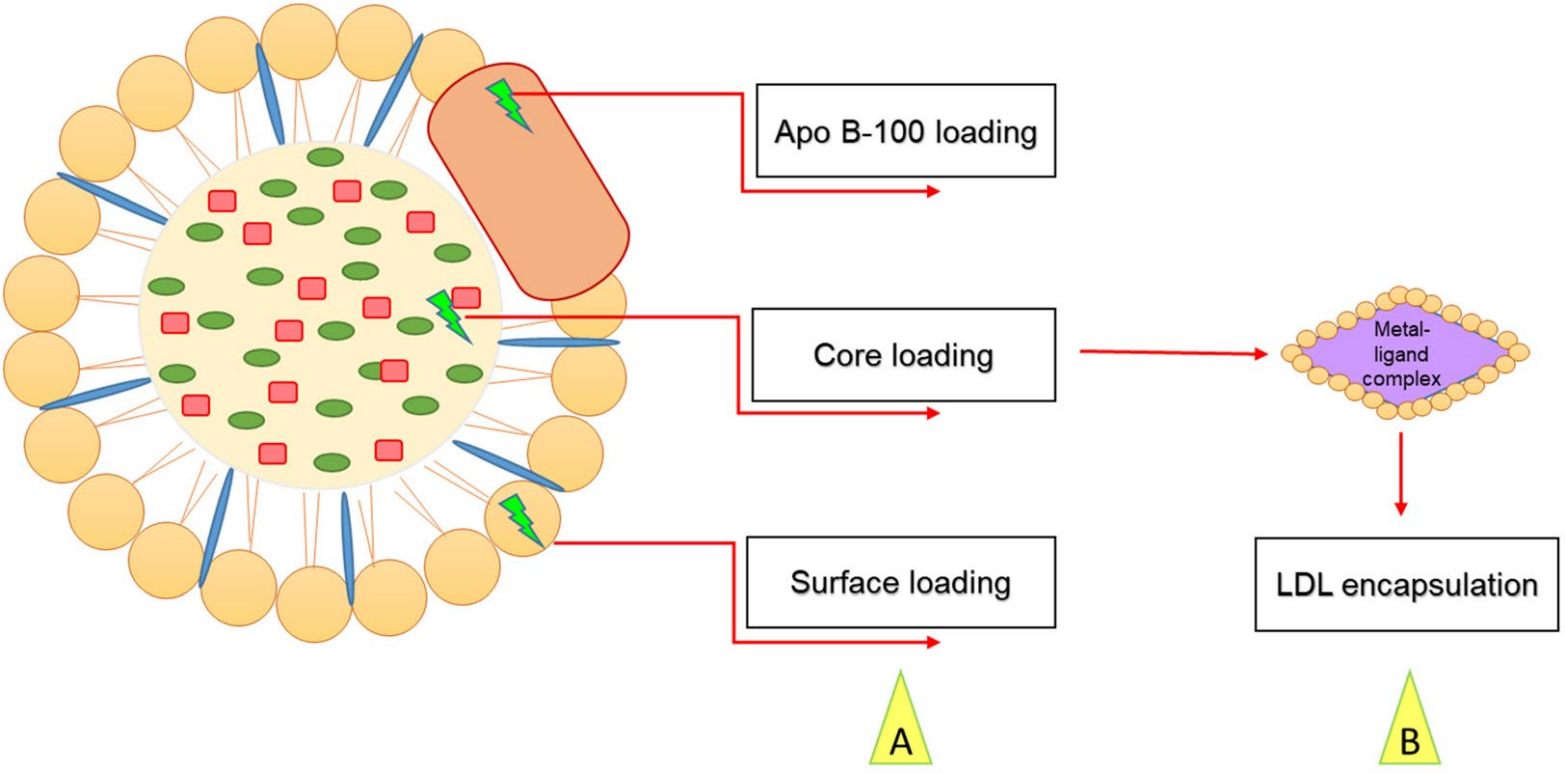
(A) Methods of loading drugs into LDL vehicles. (B) Thiosemicarbazone metal-ligand complex encapsulated into LDL core.

Many research labs recently used lipoproteins as carriers to transport imaging agents or drugs to tumors for diagnostic or therapeutic purposes (Firestone, 1994; Chen et al., 2007; Zhou et al., 2010; Nakayama et al., 2012; Huang et al., 2017; Chaudhary et al., 2019; Tang et al., 2021). LDLs are spherical particles characterized by an insoluble core consisting of cholesteryl esters and triglycerides surrounded by phospholipids and specialized apolipoprotein B-100 (Harisa and Alanazi, 2014; Alhadad et al., 2020). Zhang et al. used a complex peptide containing DNA nano LDL as an efficient drug delivery vehicle and the results showed an increase in tumor reduction and cell death (Zhang et al., 2015). Recent reports indicated the emerging role of LDL in targeting breast (Guan et al., 2019; Li et al., 2021), liver (Wang et al., 2019), and prostate cancers (Sun et al., 2017). Additionally, nano-drug delivery systems were shown effective for Alzheimer’s disease (Jaragh-Alhadad & Falahati, 2022) and target the site-specific brain with antiviral drugs (Varghese et al., 2018) to increase the delivery across the blood-brain barrier cells (Pinzón-Daza et al., 2012). Furthermore, LDL is a drug vehicle for treatments of atherosclerosis, cancer, and photodynamic therapies (Harisa & Alanazi, 2014). Drugs can be loaded into LDL particles by surface loading, core loading (Di & Maiseyeu, 2021), and apolipoprotein B-100 interactions as shown in Figure 2-A (Harisa & Alanazi, 2014; Alhadad, 2021). Loading cytotoxic drugs into LDL will provide an effective vehicle for drug transport and delivery to the specific cell because it mimics the endogenous LDL and will not be recognized as a foreign entity by the immune system (Harisa & Alanazi, 2014; Lee et al., 2015).

Nanoparticles are considered an efficient vehicle for cancer drug delivery (Chaudhary et al., 2019) and an attractive strategy for optical and biomedical applications (Abdellatif, 2020). In 2016, Abdellatif et al. used gold nanoparticles to target G-protein coupled receptors (Abdellatif et al., 2016). Recently, in 2022, Abdellatif et al. used octreotide-conjugated silver nanoparticles as an active, specific, and selective targeting of somatostatin receptor sites. The data revealed potent cytotoxic effects of the nanoparticles with MCF7 cells (Abdellatif et al., 2021b). In addition, Alhadad et al. synthesized anticancer agents and encapsulated them into nano-LDL particles, the results showed potent anticancer activity against ovarian cancer targeting heat shock protein 27 (HSP27) and human epidermal growth factor receptor 2 (HER2) (Alhadad et al., 2020). Moreover, another study used cholesterol conjugated anti-HSP27 and anti-HER2 agents encapsulated into nano-LDL particles as drug delivery enhancement against the SKOV3 cell line (Alhadad, 2021). Furthermore, Nikanjam et al. used synthetic nanoparticles to target GBM tumors, and the data described how the nano-LDLs were taken by the LDL-receptor and caused an anticancer effect (Nikanjam et al., 2007). Therefore, in this study thiosemicarbazone metal complexes were encapsulated into nano-LDL core (Figure 2-B) to benefit from the endogenous LDL metabolic pathway and reach site-specific of the cancer cell, then used in biological tests to evaluate the drug delivery strategy.

## Materials and methods

### Materials

Anticancer agents were synthesized previously in our laboratory at Kuwait University, chemically and physically characterized at the research sector project unit (RSPU) laboratories (Ali & Hasan, 2021). All the chemical reagents are commercially available with the analytical grades and ready for direct use without preparation from Merck and Sigma Aldrich.

Dulbecco Modified Eagle Medium (DMEM), fetal bovine serum (FBS), phosphate buffer saline (PBS), trypsin, L-glutathione, penicillin-streptomycin, and other supplements were supplied from the media core facility at Cleveland Clinic-Lerner Research Institute. All antibodies were from cell signaling technology, while the β-actin housekeeping gene was purchased from SANTA CRUZ BIOTECHNOLOGY. WST-1 kit was purchased from ABCAM. LDL depleted serum was purchased from Sigma Aldrich.

## Methods

### Agents’ particle size measurements

Thiosemicarbazone ligand complex was not able to disperse in water, DMF, DMSO, Toluene, Methanol, or Ethanol. On the other hand, all thiosemicarbazone metal-ligand complexes were dispersed easily with a suitable solvent such as chloroform and toluene. In-depth, 100 µL of the LDL-particles encapsulated ligand complex mixed with two µL of DMSO and sonicated five minutes before the measurement by the Zeta sizer device. These steps were repeated exactly for the LDL particles encapsulated with metal-ligand complexes. The size measurements were done using a zeta sizer at pH seven (Nano ZS-Malvern Paralytical Ltd, UK) at the Kuwait University-RSPU facility (project GS01/03).

### Encapsulation of low-density lipoprotein with thiosemicarbazone metal-ligand complexes

**Figure 3.**
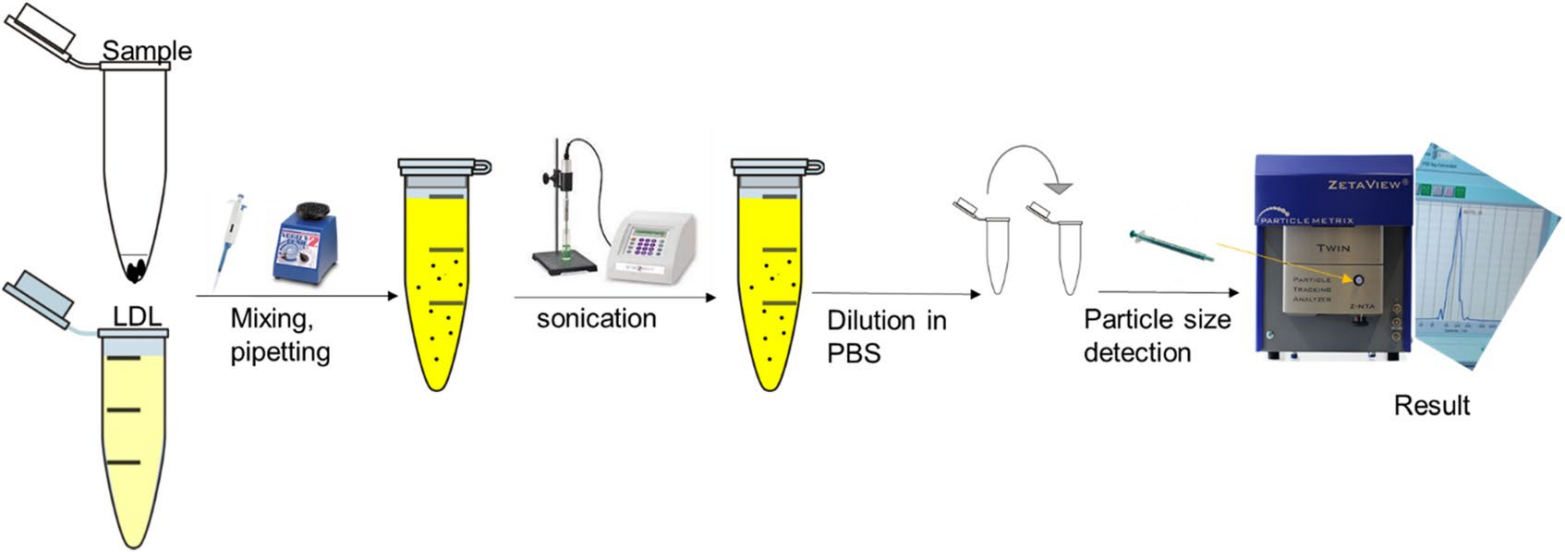
Encapsulation method and particle size measurement.

The structure of the commercially available LDL-depleted serum is like the native LDL (Sigma Aldrich). Therefore, 50 µl of LDL-particles was used for encapsulation with a five µl ligand (5:1) ratio. LDL-particles and the ligand were mixed by simple pipetting, vortexing, and sonicating to break down the large LDL-particles into small LDL-particles (Poly-Tron dispersing and mixing device made in Switzerland by Kinematica AG), and the sample was left overnight to let LDL-particles reform, reconstruct again surrounding the ligand before any use as shown in Figure 3. These steps were repeated for all metals-ligand complexes (Ni, Pt, Pd, VO, Mn, Cu, CuClO_4_, CuAg) and samples were stored at 4 °C before measuring their particle sizes and left for one month before cytotoxicity assessment (Harisa & Alanazi, 2014; Lee et al., 2015; Alhadad et al., 2020; Alhadad, 2021).

### Measurement of the size distribution after LDL encapsulation

Briefly, after encapsulation, PBS was used as blank and used for washing between each sample run in the Zeta view device. First, ten µl of LDL was used as free control vehicle to measure background size in 1000 µ l of PBS. This step was repeated for all LDL vehicles encapsulated with metal-ligand complexes at pH seven. The size distribution was measured by Zeta view-Particle Tracking Analyzer-Flow cytometry-core facility at the Lerner Research Institute at Cleveland Clinic (Alhadad et al., 2020; Alhadad, 2021).

### Cell culture

A breast cancer (MCF7) cell line was purchased from the cell culture core at Cleveland Clinic-Lerner Research Institute. Lung cancer (A549) cells were a gift from Dr. Robert Silverman from Lerner Research Institute. Prostate cancer (C42-positive androgenic receptor) cells were a gift from Dr. Nima Sharifi from Lerner Research Institute. Cells were maintained in DMEM media supplemented with 10% FBS, 1% penicillin/streptomycin, and 1% L-Glutathione, and incubated in humidified air with 5% CO_2_. FBS is inactivated at a 37 °C water bath for 30 minutes before use (Alhadad et al., 2020; Alhadad, 2021).

### Cytotoxicity studies

The in-vitro cytotoxicity assay was carried out based on the manufacturer’s protocol provided by WST-1 assay Kit (ab65473 Abcam, USA). Briefly, 5 × 10^4^ cells/well/ml from the MCF7, A549, and C42 cell lines were seeded on 96-well plates and the next day treated with various LDL-particles encapsulated metal-ligand complexes in a dose-dependent manner and incubated for 48 hr (Alhadad et al., 2020; Alhadad, 2021). WST-1 reagent was added, and absorbance was measured using a plate reader (SoftMax Pro 9.0 Flex Station-Molecular devices). The assay was performed in quadruplicate concentrations.

### Statistical analysis

Statistical analysis data were performed and analyzed by Graph Pad Prism software and the results normalized to controls by nonlinear regression analysis. Cytotoxicity data were presented as mean ± standard deviation (SD) from three independent tests.

### Western blot assay

Cells were seeded into six-well plates and incubated overnight before treatment with agents (one mM) for 48 hr. The next day, cells were washed with PBS, harvested, and lysed with mammalian protein extraction reagent (MEMBER) buffer. The cell suspension was centrifuged at 12,000 rpm for 15 min and the supernatants were collected and protein determination was performed using the Bradford assay. An equal amount of protein was loaded into western blot gel (NuPAGE 10% Bis-Tris Gel) before transfer onto a nitrocellulose membrane. The membrane was blocked in Odyssey blocking buffer (LI-COR) for one hour at room temperature and subsequently overnight at 4 °C in the following primary antibodies tubulin (1:1000 dilution) and Β-actin (1:10,000 dilution). Membranes were washed with PBST (phosphatized buffered saline containing 0.1× Tween 20) and then incubated with anti-mouse anti-rabbit antibodies. Blots were imaged with Odyssey infrared imaging system in both 700 and 800 nM channels (imaging core-Lerner Research Institute. Cells’ pictures were taken using the microscope (EVOS FL life technology) after 48 hr. of the treatment (Alhadad et al., 2020; Alhadad, 2021).

## Results

### Particle size measurements before and after encapsulation with LDL

Thiosemicarbazone metal complexes were dispersed in chloroform and toluene solvents as shown in Table 1, to measure the chemical complexes’ particle size before encapsulation. The results revealed that the agent’s diameters were in the nano levels except for the ligand complex which was not able to disperse in any solvent as mentioned previously in the methods section because of the absence of the ionic interactions between the metal and the ligand. The data showed that the smallest nano-particle size sequentially is CuAg-L< Mn-L< VO-L< Ni-L< Cu-L< Pt-L< CuClO_4_-L< Pd-L complex.

**Table 1.** Particle size measurements for thiosemicarbazone metal-ligand complexes before LDL-particles’ encapsulation.

Complex	Particle size nm	S.D.	Solvent
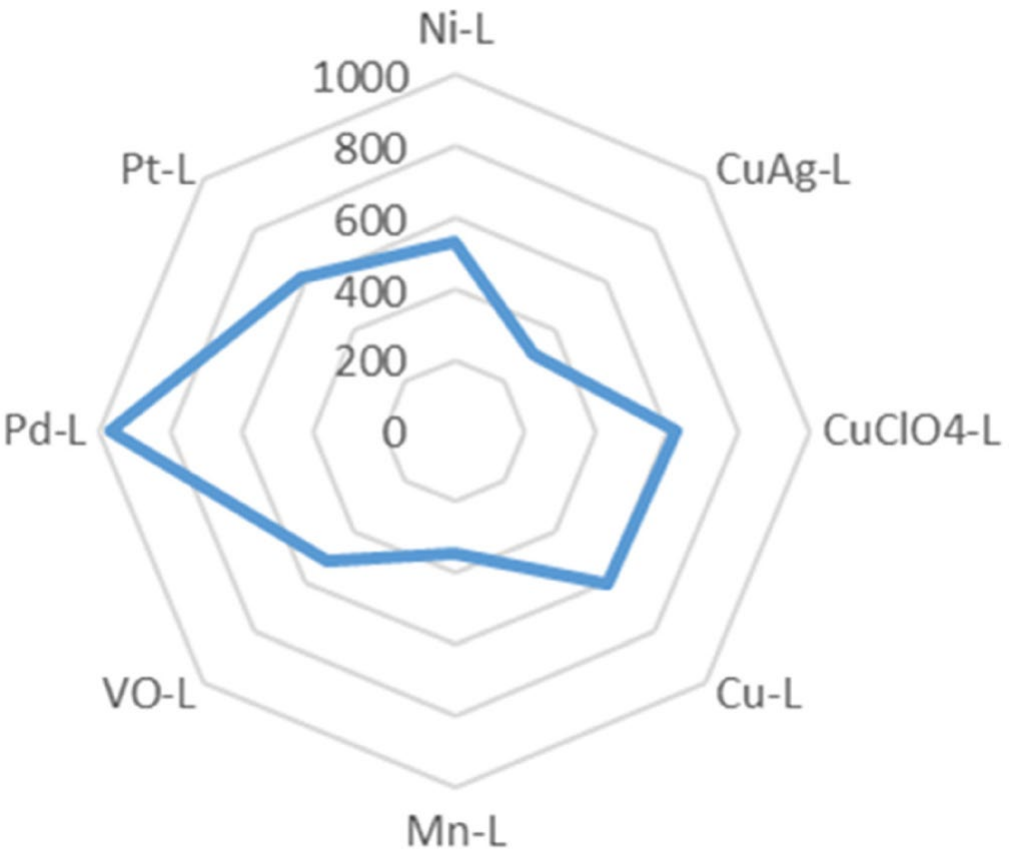			
Ligand	Was not able to disperse in any solvent.		
Ni-L	535.2	18.04	Chloroform
Pt-L	614.2	66.93	Toluene
Pd-L	973.5	250.3	Toluene
VO-L	514.9	35.65	Toluene
Mn-L	342.0	6.607	Toluene
Cu-L	609.5	21.06	Toluene
CuClO_4_-L	621.3	66.99	Toluene
CuAg-L	309.4	41.05	Toluene

Sample % intensity is 100 for all agents.

After encapsulation of metal-ligand complexes into LDL particles, samples were left to recover, and reconstitute again, and then particle sizes were measured by a zeta sizer. The plain LDL and metals ligand complexes range between 56 nm to 91 nm in diameter. The zeta potential data showed the size of the free LDL particles is 56.80 nm, LDL particles encapsulated with ligand have a size of 66.25 nm, LDL particles encapsulated in nickel-ligand complex have the size of 75.70 nm, LDL particles encapsulated in the platinum-ligand complex have the size of 77.70 nm, LDL particles encapsulated VO-ligand complex have the size of 91.15 nm (VO-ligand complex exhibited the highest particle size), LDL particles encapsulated copper-ligand complex have the size of 89.45 nm, LDL particles encapsulated Mn-ligand complex have the size of 83.30 nm, LDL particles encapsulated Pd-ligand complex have the size of 86.25 nm, LDL particles encapsulated CuClO_4_-ligand complex have the size of 71.35 nm, LDL particles encapsulated CuAg-ligand complex have the size of 76.85 nm, as summarized in Table 2. Generally, the LDL particles encapsulated ligand exhibited a larger size than the plain LDL particles. In addition, the data revealed that the particle size of metal-ligand complexes were larger than the ligand itself because the main ligand structure contains central metal with different geometries and binding interactions which makes the complex size bigger. In summary, the size of metals ligand complexes was larger than the plain LDL, proof of a successful encapsulation strategy (Table 2) (Alhadad et al., 2020; Alhadad, 2021).

**Table 2.** Data collection from Zeta potential nanoparticles’ diameters.

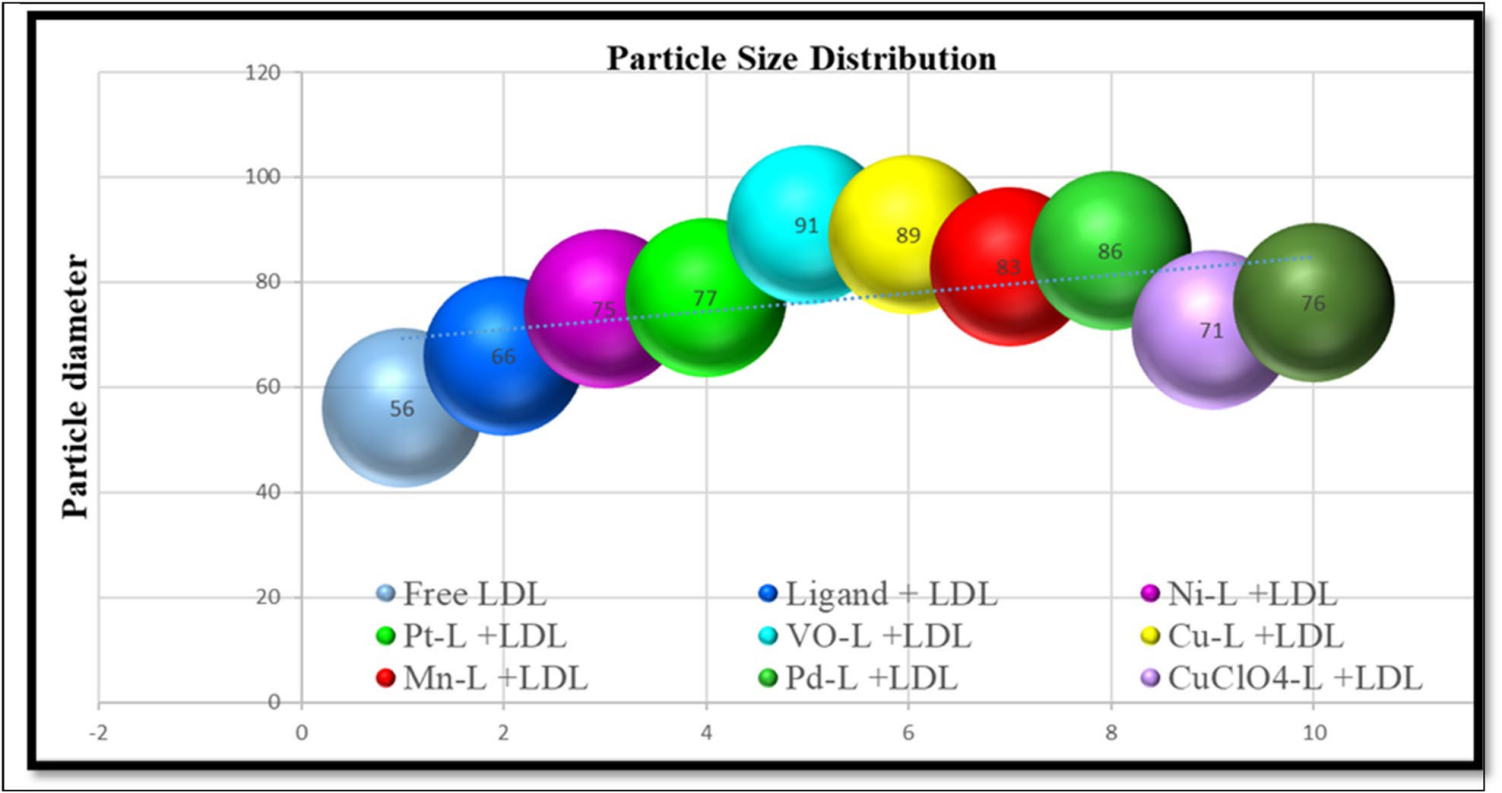				
Particle	Diameter/nm	Percentage %	Mean ± SD	Median X50
1—Free LDL	56.80^a^	98.50	62.15 ± 23.45	55.60
2—Ligand + LDL	66.25^b^	100.0	70.55 ± 24.15	64.20
3—Ni-L + LDL	75.70^c^	90.85	78.95 ± 36.55	71.55
4—Pt-L + LDL	77.70^c^	97.25	78.50 ± 33.85	71.40
5—VO-L + LDL	91.15^c^	96.80	86.45 ± 38.00	79.05
6—Cu-L + LDL	89.45^c^	96.60	91.80 ± 39.60	84.75
7—Mn-L + LDL	83.30^c^	80.15	75.80 ± 39.20	76.75
8—Pd-L + LDL	86.25^c^	94.80	88.30 ± 41.85	80.65
9—CuClO_4_-L + LDL	71.35^c^	79.85	80.60 ± 35.00	69.60
10—AgCu-L + LDL	76.85^c^	97.00	76.25 ± 32.75	68.20

Data presented from the average of two independent runs.

^a^Plain vehicle = LDL (control).

^b^Vehicle containing the ligand, particle size increased based on the control.

^c^Vehicle containing the metal-ligand, particle size increased based on the control.

*All vehicles containing ligand and metal-ligand complexes, particle size significantly increased.

### In-vitro cytotoxicity assessments for breast, lung, and prostate cancer cells

**Figure 4.**
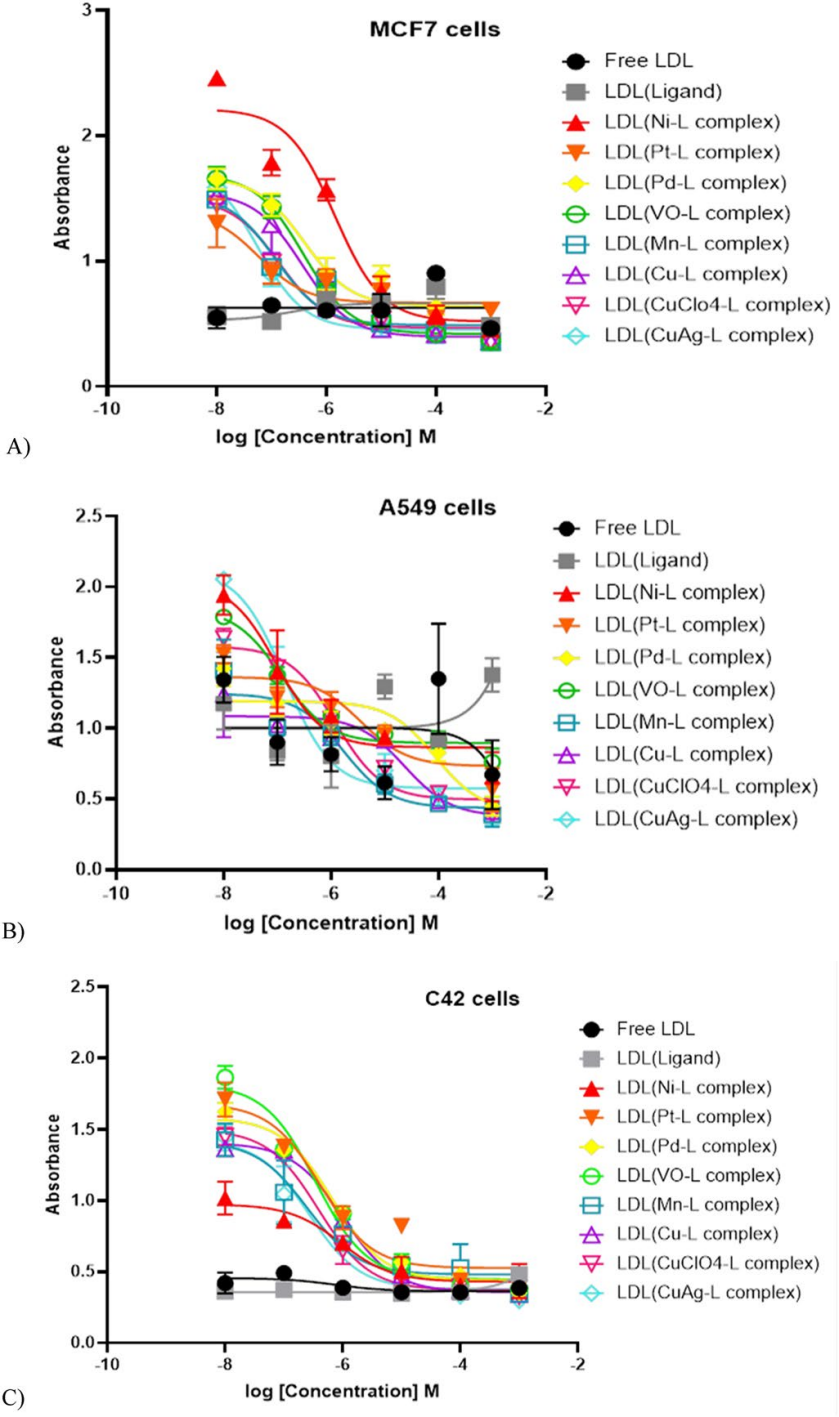
(A) MCF7, (B) A549, and (C) C42 cell lines showed excellent cancer growth inhibition.

After the one-month LDL-particles encapsulation, samples were tested for the cytotoxicity effects by dose-dependent treatments (Alhadad et al., 2020; Alhadad, 2021). The IC_50_ values were calculated and reported in (Table 3). The data revealed that all cell lines showed elevated growth inhibition (Figure 4). The results revealed plain LDL particles do not affect cell growth inhibition. Alternatively, cells treated with LDL particles encapsulated with the ligand showed significant cell growth inhibition at one µM. The IC_50_ values of metal-ligand complexes showed more growth inhibition than treatment with the plain LDL particles that contained the parent ligand without a metal-ligand complex. MCF7 IC_50_ data ranges between 1.18–6.56 µM, A549 between 1.17–9.66 µM, and C42 between 1.01–6.62 µM. In-depth, LDL containing (CuClO_4_-ligand complex) was the most potent agent affecting MCF7 cell growth inhibition, while LDL containing (Cu-ligand complex) was the strongest agent affecting cancer cell growth of both A549 and C42 cell lines. Furthermore, LDL encapsulated (CuAg-ligand complex) showed the same range of growth inhibition in all cell lines with an IC_50_ value average of two µM. Overall, LDL encapsulated thiosemicarbazone metal-ligand complexes targeted C42 >MCF7 > A549 cells.

**Table 3.** IC_50_ values of MCF7, A549, and C42 cancer cell lines treated with LDL-particles encapsulated thiosemicarbazone metal-ligand complexes.

Encapsulation	MCF7 IC_50_/1 µM	A549 IC_50_/1 µM	C42 IC_50_/1 µM
Plain LDL	No effect	No effect	No effect
Ligand	Control cell growth	Control cell growth	Control cell growth
Ni-Ligand complex	2.40 ± 1.91^****^	9.66 ± 1.17*	1.47 ± 0.53**
Pt-Ligand complex	6.56 ± 0.64***	3.74 ± 0.87***	4.22 ± 1.15^****^
Pd-Ligand complex	3.89 ± 1.03**	8.80 ± 0.78**	6.62 ± 1.14^****^
VO-Ligand complex	6.61 ± 1.14***	1.18 ± 0.95**	3.46 ± 1.37^****^
Mn-Ligand complex	1.37 ± 1.03^****^	5.39 ± 1.01***	2.48 ± 0.93^****^
Cu-Ligand complex	3.59 ± 1.15^****^	1.17 ± 0.55**	1.01 ± 0.89^****^
CuClO_4_-Ligand complex	1.18 ± 1.05^****^	2.18 ± 0.86***	3.90 ± 1.13^****^
CuAg-Ligand complex	2.52 ± 1.76^****^	2.60 ± 1.39^****^	2.69 ± 1.05^****^

MCF7: breast cancer, A549: lung cancer, C42: prostate cancer cell line.

*P-value.

**Figure 5.**
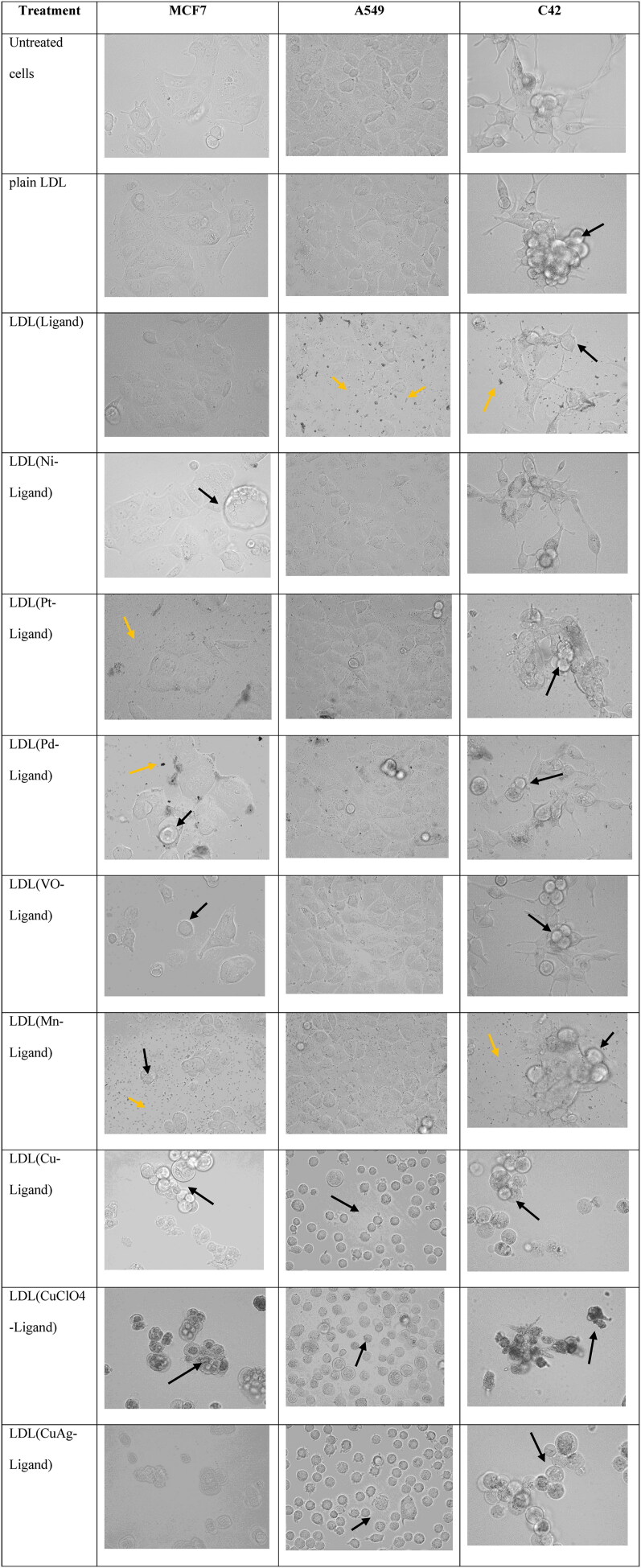
MCF7, A549, and C42 cells morphology after 48 hr. treatment with one mM drug concentrations. The untreated cell lines and treated with plain LDL particles showed normal cell morphology. LDL particles encapsulated with copper complexes were the most potent agents in breast, lung, and prostate cancer cell growth inhibition. Black arrows showed cell death after treatment with LDL particles encapsulation thiosemicarbazone metal-ligand complexes while yellow arrows showed cell death and agents precipitation.

The cells’ morphology by bright field microscopy took after one month of the encapsulation to check the stability and efficacy of the LDL particles when treated with cancer cells. The data showed various levels of cell death as well as sometimes precipitation in the cell culture medium (Figure 5) with no large aggregation which proves the stability of the LDL particles. All the cell lines were untreated, treated with free LDL particles, and treated with LDL particles encapsulated with metal-ligand complexes. The untreated cells were used as a control. The cells treated with plain LDL particles showed no effect on the growth of the cells in the three cell lines. Cells treated with LDL particles encapsulated with ligand showed cell death and ligand precipitation in the media. Cells treated with LDL particles encapsulated metal-ligand complexes showed different levels of cancer growth inhibition. In sum, LDL particles encapsulated thiosemicarbazone metal-ligand complexes showed effective cancer cell growth inhibition.

### Western blot assay-agents targeting tubulin protein

**Figure 6.**
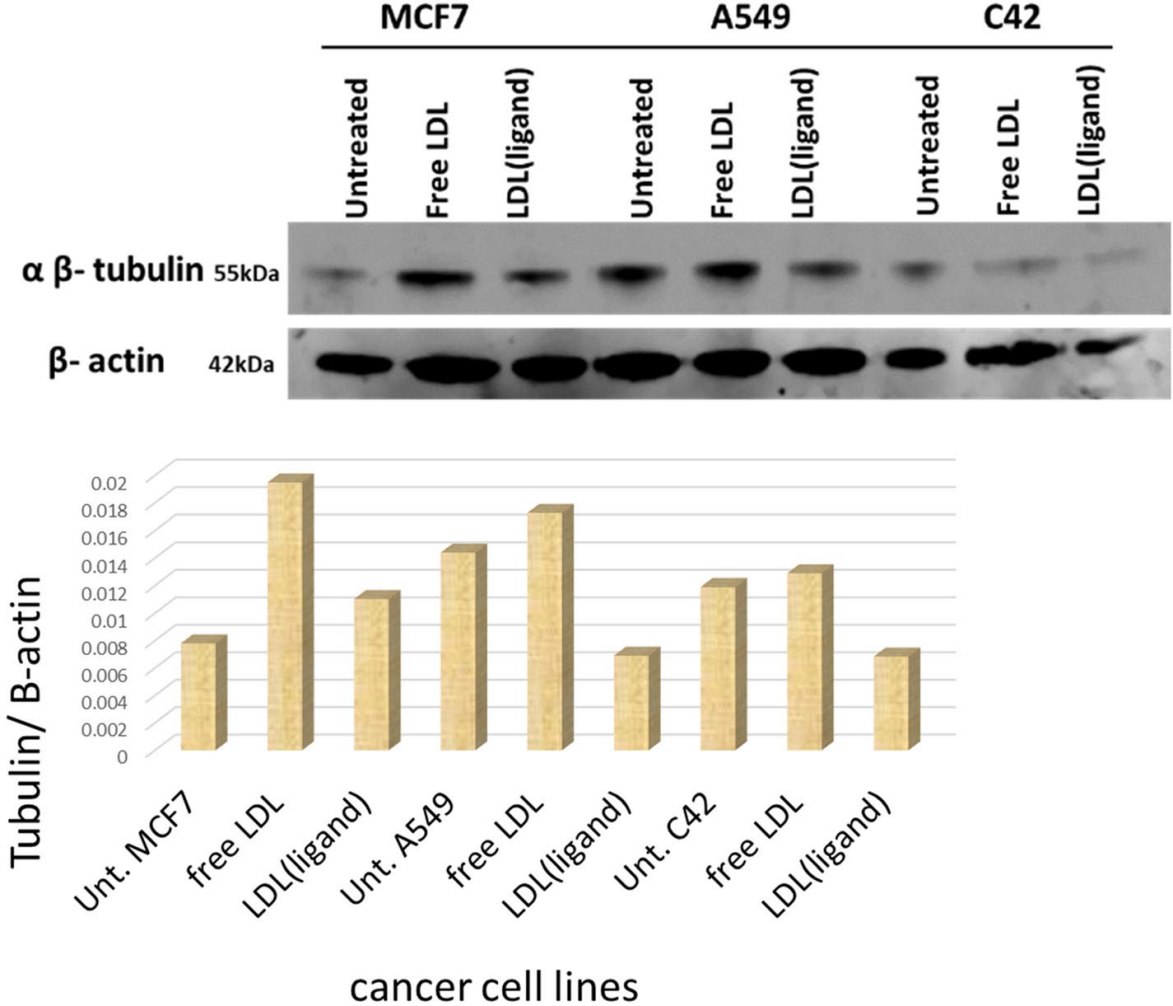
Three cancer cell lines were untreated, treated with free LDL particles, and treated with LDL particles encapsulated with the ligand. Cells treated with LDL particles encapsulated with ligand were potent in targeting tubulin in cancer cell lines and reduced its expression.

**Figure 7.**
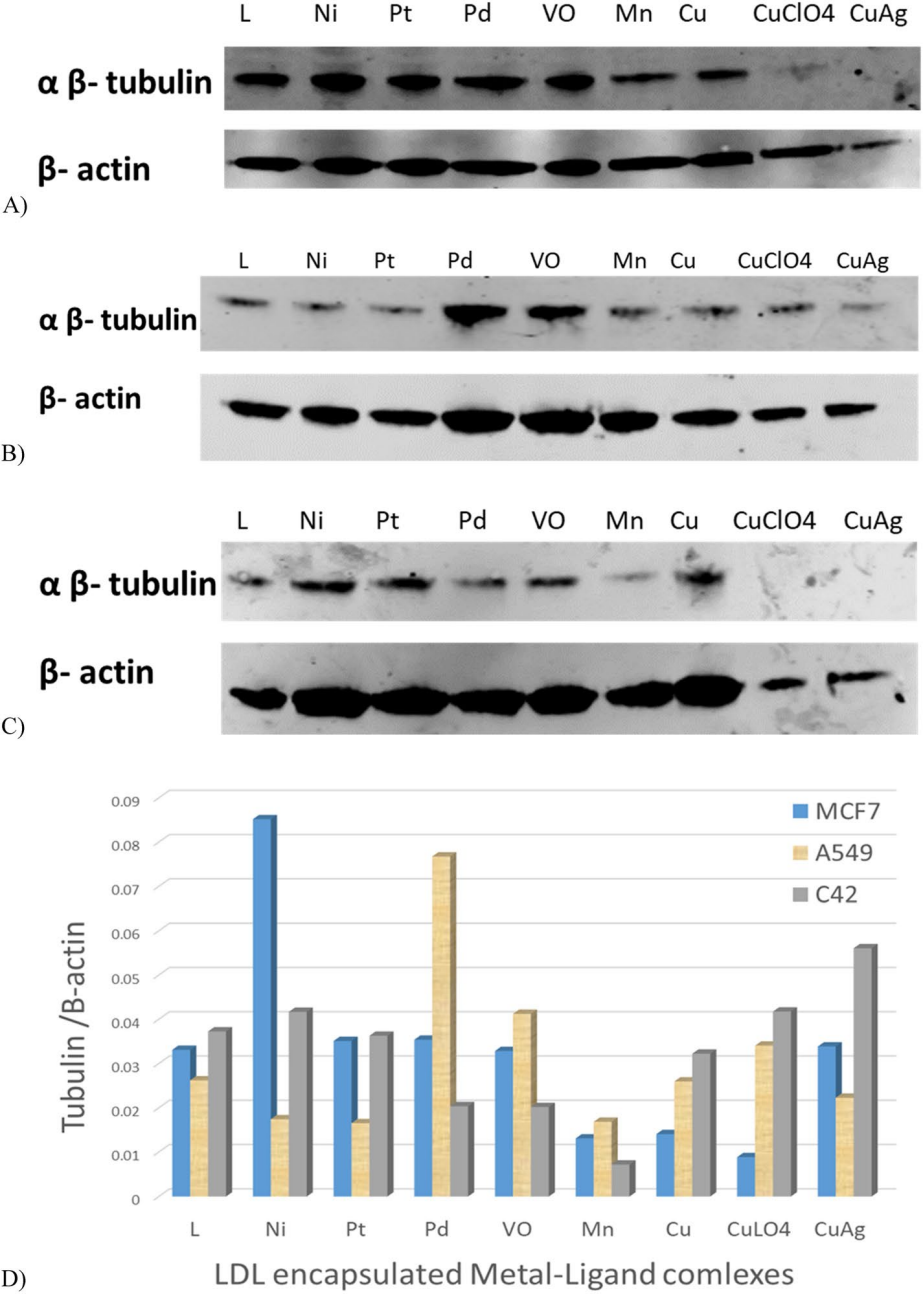
LDL particles encapsulated thiosemicarbazone metal-ligand complexes treated with (A) MCF7, (B) A549, (C) C42, and (D) gel quantification bands. The LDL particles encapsulated metal-ligand complexes showed excellent cell growth inhibition by targeting tubulin and reducing its expression.

Tubulin protein levels were assessed by western blot analysis. Figure 6 showed the three cancer cell lines untreated, treated with free LDL particles, and treated with LDL particles encapsulated with the ligand. Analysis showed that MCF7, A549, and C42 treated with plain LDL particles have high tubulin levels, whereas cells treated with LDL particles encapsulated with ligand had reduced levels. Treatments with Mn-L and copper-L complexes were the most potent agents that reduced tubulin expression in MCF7 cells (Figure 7-A). Secondly, the LDL particles encapsulated ligand and its metal complexes including Ni-L, Pt-L, Mn-L, Cu-L, CuClO_4_-L, and CuAg-L complexes were potent agents in A549 cells treatment (Figure 7-B). Third, LDL-particles encapsulated Mn-L and Cu-L, CuClO_4_-L, and CuAg-L complexes were the most potent agents in C42 cell treatments by reducing tubulin expression (Figure 7-C) Generally, LDL particles encapsulated Mn-L and copper-L complexes showed excellent cancer cell growth reduction in the three tested cancer cell lines as shown in the gel quantification bands Figure 7-D.

## Discussion

Nano LDL-particles are raising more attention these days for their potential use as an active drug delivery strategy for cancer treatments (Abdellatif, 2020; Abdellatif et al., 2021b; Alhadad, 2021). LDL proved to be a very stable platform, highly selective (Abdellatif et al., 2016; Zhang and Huang, 2017; Abdellatif et al., 2021b) for anticancer drug delivery (Lee et al., 2015; Alhadad et al., 2020; Alhadad, 2021). It was observed that cancer patients have low cholesterol levels due to the uptake of LDLs by the tumors, cancer cells need a high amount of cholesterol for the new cell membrane synthesis because cholesterol is an important part of the lipid bilayer (Firestone, 1994; Nikanjam et al., 2007; Chaudhary et al., 2019).

Previously, thiosemicarbazone ligand and metal-ligand complexes were synthesized and fully characterized at Kuwait University laboratory by x-ray crystallography, proton and carbon NMR, IR, molecular weight, molar conductivity, color, melting point, partial elemental analysis, XRD, electronic and magnetic measurements, DSC and TGA thermal analysis, and the bacteria and fungi activities (Ali & Hasan, 2021). In 2021, Ali et al. proved that the ligand has an orthorhombic crystal system, and it is a chelating agent that acts as both neutral and dibasic pentadentate (N3S2); neutral and dibasic tetradentate (N2S2). On the other hand, metal-ligand complexes are potent chelating agents and exhibited octahedral (Ni-L complex), square planner (Pt-L and Pd-L complexes), and square-pyramid (VO-L, Mn-L, Cu-L, CuClO_4_-L, and CuAg-L complexes) geometrical arrangement (Ali & Hasan, 2021). It is important to mention that the transition metals increase the stability of the complexes (Abdellatif et al., 2016; Abdellatif, 2020; Ali & Hasan, 2021) and the positive metal ion toxicity to the living organism (Shaw, 1961; Paterson & Donnelly, 2011). Thiosemicarbazone is known for its strong NNS chelating agent, characterized by anticancer activity, and this activity increase when metals exist in the structure which has progressed to human clinical trials (Paterson & Donnelly, 2011; Shakya & Yadav, 2020).

Based on that, thiosemicarbazone agents were used as our anticancer agent, and the size of its metal-ligand complexes was measured by a Zeta sizer and the diameter was in the nanometer levels. It is known that the small nanoparticles exhibit important physicochemical and biological properties and benefit nanomedicine for the treatment of cancer (Abdolmaleki et al., 2020). This step was followed by the encapsulation strategy of the anticancer agents into LDL particles, which also were in the nano levels. The zeta potential data showed that LDL particles diameter containing metal-ligand complexes were bigger than the LDL particles containing the ligand, which is also bigger than the plain LDL particles. This is due to the interactions and geometry of the central metal ion with the ligand which increase the stability of the complex (Ali & Hasan, 2021). Any drug delivery strategy starts with the agents’ stability both at 37 °C for treatment and at 4 °C for storage which proven previously (Alhadad et al., 2020; Alhadad, 2021). These nano LDL-particles enhance the therapeutic index by diminishing their toxicity against physiological tissues and achieving controlled therapeutic levels to cancer cell site-specific (Alhadad et al., 2020; Alhadad, 2021) and lower drug concentrations intake (Verma et al., 2018). The LDL-particles encapsulated thiosemicarbazone metal-ligand complexes left for one month before use, the images showed uniform LDL-particles with no aggregation or morphological changes. Abdellatif et al. confirmed that silver nan-particles physical stability reached up to three months at the following temperatures 25 ± 0.5 °C and 4.0 ± 0.5 °C (Abdellatif et al., 2021a). These nano-sized particles benefit cancer treatments by having efficient encapsulation and efficient drug delivery to cancer cells.

Alhadad et al. designed and encapsulated anticancer dual HSP27 and HER2 inhibitors into low-density lipoprotein to target the SKOV3 ovarian cancer cell line and the IC_50_ results were in the 22.5 µM level (Alhadad et al., 2020). Lee et al. targeted both MCF7 and A549 cancer cell lines with drug LDL-particles and the results of the IC_50_ value were potent between 6.8–9.1 µM (Lee et al., 2015). In 2016, new naphthalene substituted thiosemicarbazone derivatives were synthesized and evaluated against an A549 cancer cell line with an IC_50_ value of 31.25 μg/mL when compared with cisplatin (IC_50_ = 16.28 μg/mL) (Altıntop et al., 2016). In addition, Shao et al. synthesized the thiosemicarbazone Cu(II) complex, and its IC_50_ value reached 11.2 ± 0.9 μM against Hep G-2 cells (Shao et al., 2014). Moreover, Copper(II), nickel(II), palladium(II), and platinum(II) complexes of ortho-naphthoquinone thiosemicarbazone were synthesized, characterized, and evaluated using MCF7 cells. Results revealed that the nickel compound exhibits the lowest IC_50_ value 2.25 μM (Xavier et al., 2020). In this study, the cytotoxicity assessments of the LDL-particles encapsulated anticancer agents against MCF7, A549, and C42 cancer cells were below 6.56 µM, 9.66 µM, and 6.42 µM respectively, and these are perfect results compared to the previous studies. This proved that the LDL-particles internalized into the cell through LDL-receptor and the anticancer agent reach the site-specific in the cancer cell and caused cell death (Alhadad et al., 2020; Alhadad, 2021).

Tubulin protein expression for the MCF7, A549, and C42 cell lysates showed no effect when the cells were untreated/and treated with the plain LDL particles. On the other hand, tubulin expression was reduced when the cells were treated with the LDL-particles containing the ligand, this expression was reduced more when the cells were treated with the LDL-particles containing positive metal-ligand complexes. Xavier et al. synthesized and studied the quantum mechanical modeling, biomolecular interaction, and in-vitro anticancer activity against A549 and HepG2 cancer cell lines with IC_50_ values of 0.051–0.189 µm and 0.042–0.136 µm respectively, targeting tubulin activity by thiosemicarbazone (Xavier et al., 2020).

**Figure 8.**
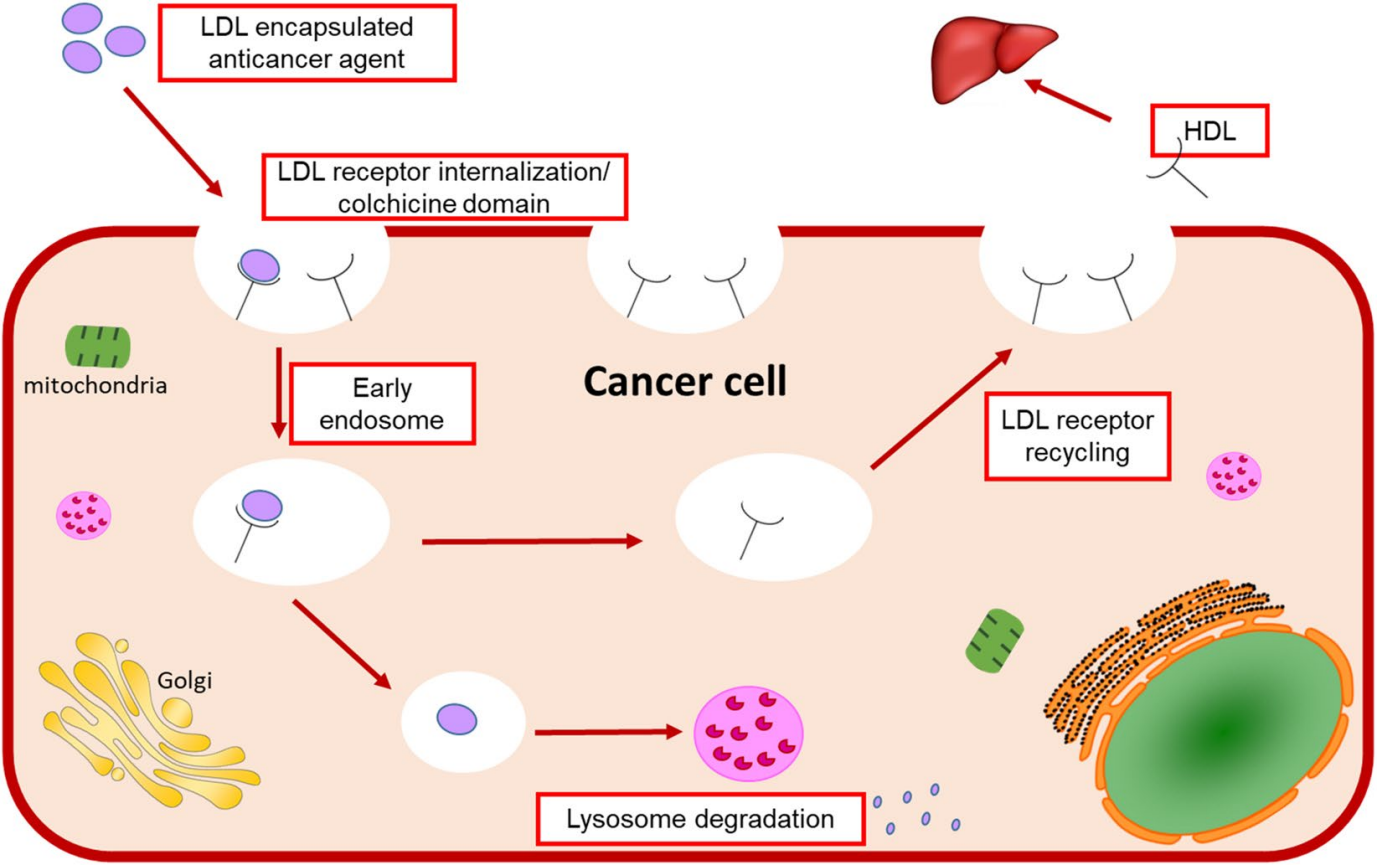
Schematic representation of LDL particles encapsulated thiosemicarbazone metal-ligand complex targeting LDL receptor to internalize cancer cells.

In summary, this study accomplished the goal by the benefits of the particle’s nano-size, chelating property, geometry, encapsulating anticancer agents into LDL particles by core loading strategy, and the LDL particles were at the nano level. Then, these nanoparticles bind to the cells’ LDL-receptors and internalize and then release the anticancer thiosemicarbazone metal-ligand complexes inside cancer cells. This is causing high cytotoxic activity, reducing tubulin expression, therefore mimicking the natural LDL particles’ metabolic pathway as shown in the systematic representation in Figure 8.

## Conclusion

Anticancer drugs encapsulated into nano-LDL particles mimic the natural lipid metabolic pathway. In this study, LDL-particles encapsulated thiosemicarbazone metal-ligand complexes were used as an active drug delivery vehicle for cancer treatment targeting tubulin protein in the breast (MCF7), lung (A549), and prostate (C42) cancer cells. After encapsulation of the agents in the core of the LDL particles, the Zeta potential data showed an increase in the nano LDL particles’ size compared to the plain LDL particles, proving the loading strategy. The cytotoxicity assessments reported potent cancer cell growth inhibition in all cell lines studied. Western blot assay showed LDL particles encapsulated ligand and metal-ligand complexes led to a reduction in tubulin expression in tested cancer cell lines. The data observed from the Zeta potential, morphology images, MTT, and western plot assays were parallel and confirmed the potency of our agents in the reduction of cancer cell growth, especially LDL particles encapsulated with copper-L complexes. Overall, these findings confirmed that the LDL particles encapsulation strategy mimics the LDL’s natural metabolic pathway and is used as an excellent vehicle for active drug delivery for cancer therapy.
